# Assessing the quality and accuracy of national immunization program reported target population estimates from 2000 to 2016

**DOI:** 10.1371/journal.pone.0216933

**Published:** 2019-07-09

**Authors:** Lena A. Stashko, Marta Gacic-Dobo, Laure B. Dumolard, M. Carolina Danovaro-Holliday

**Affiliations:** 1 Strategic Information Group, Expanded Program on Immunization (EPI), Department of Immunization, Vaccines and Biologicals (IVB), World Health Organization, Geneva, Switzerland; 2 Department of International Health, Global Disease Epidemiology and Control Program, Johns Hopkins Bloomberg School of Public Health, Baltimore, Maryland, United States of America; London School of Hygiene and Tropical Medicine, UNITED KINGDOM

## Abstract

**Background:**

A common means of vaccination coverage measurement is the administrative method, done by dividing the aggregated number of doses administered over a set period (numerator) by the target population (denominator). To assess the quality of national target populations, we defined nine potential denominator data inconsistencies or flags that would warrant further exploration and examination of data reported by Member States to the World Health Organization (WHO) and UNICEF between 2000 and 2016.

**Methods and findings:**

We used the denominator reported to calculate national coverage for BCG, a tuberculosis vaccine, and for the third dose of diphtheria-tetanus-pertussis-containing (DTP3) vaccines, usually live births (LB) and surviving infants (SI), respectively. Out of 2,565 possible reporting events (data points for countries using administrative coverage with the vaccine in the schedule and year) for BCG and 2,939 possible reporting events for DTP3, 194 and 274 reporting events were missing, respectively. Reported coverage exceeding 100% was seen in 11% of all reporting events for BCG and in 6% for DTP3. Of all year-to-year percent differences in reported denominators, 12% and 11% exceeded 10% for reported LB and SI, respectively. The implied infant mortality rate, based on the country’s reported LB and SI, would be negative in 9% of all reporting events i.e., the country reported more SI than LB for the same year. Overall, reported LB and SI tended to be lower than the UN Population Division 2017 estimates, which would lead to overestimation of coverage, but this difference seems to be decreasing over time. Other inconsistencies were identified using the nine proposed criteria.

**Conclusions:**

Applying a set of criteria to assess reported target populations used to estimate administrative vaccination coverage can flag potential quality issues related to the national denominators and may be useful to help monitor ongoing efforts to improve the quality of vaccination coverage estimates.

## Introduction

One of the goals of the Decade of Vaccines, and its monitoring framework the Global Vaccine Action Plan (GVAP) endorsed by all Member States of the World Health Organization (WHO) in 2012, is to meet vaccination coverage targets in every region, country and community [1]. By 2020, this means reaching 90% national coverage of all recommended vaccines in all countries’ national immunization program [1]. Coverage estimates are monitored globally through the WHO and United Nations Children’s Fund (UNICEF) Joint Reporting Form (JRF) process, [2] where countries report vaccination coverage and other immunization data annually. A critical component of estimating vaccine coverage is an accurate corresponding target population estimate, given that most countries use the administrative method of dividing the vaccine doses administered by the target population [1]. Estimates of target populations are often live births (LB) for vaccines recommended at birth or surviving infants (SI) for vaccines recommended between 6 weeks and <1 year of age [3]. To better monitor progress towards GVAP national coverage estimates, it is necessary to monitor the underlying data, which includes critically assessing trends in the completeness, consistency, and accuracy of JRF reported target populations used as the denominator to calculate vaccination coverage [1].

Previous studies have suggested low accuracy in administrative vaccination coverage denominators, with vaccination coverage often exceeding 100%, unrealistic year-to-year fluctuations, and disease outbreaks in areas reporting high coverage [3]. Studies comparing the United Nations Population Division (UNPD) projections of LB and SI to administrative estimates have suggested a tendency for administrative target populations to be lower than UNPD estimates [4]. Another study found that coverage levels of a third dose of a diphtheria-tetanus-pertussis containing vaccine (DTP3) were higher when calculated using JRF target populations as the denominator compared to coverage estimates re-calculated using UNPD target population estimates [5].

**Fig 1:**
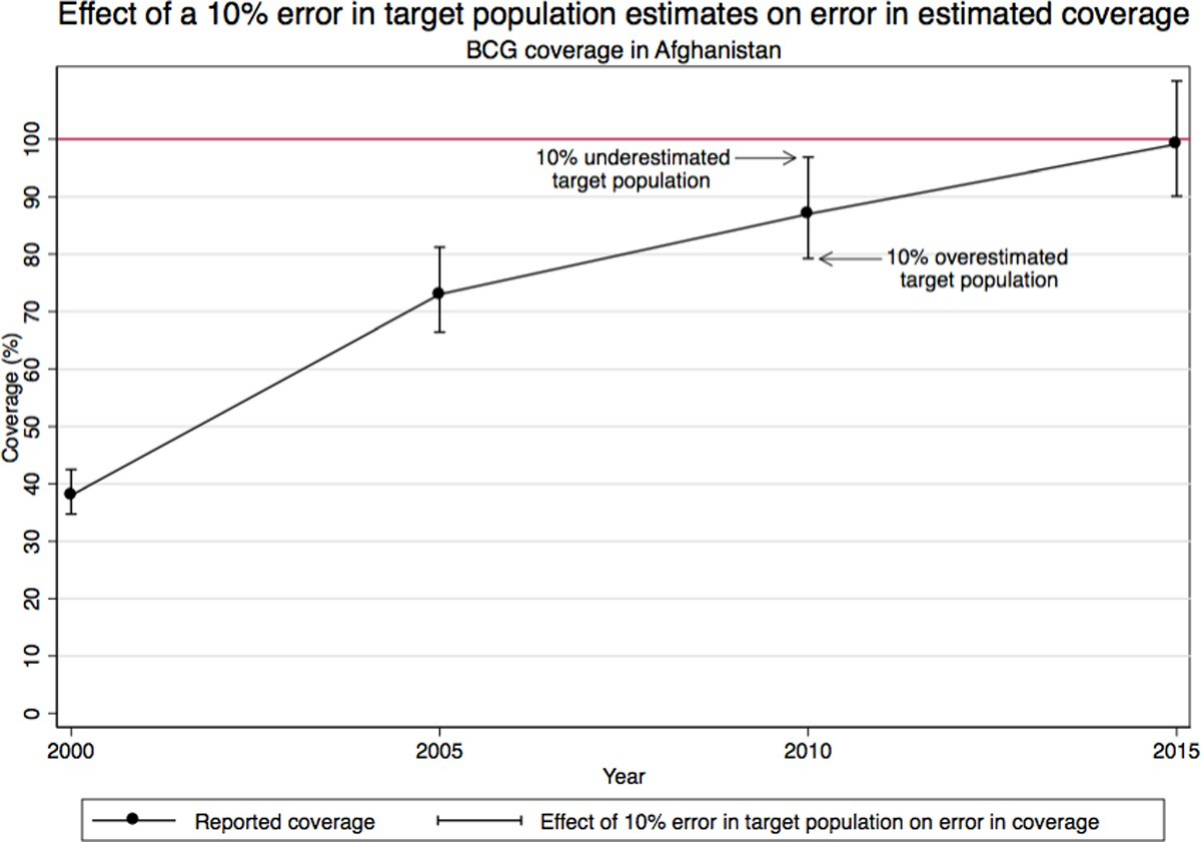
Effect of a 10% error in reported target population estimates on error in estimated coverage as coverage increases, using Afghanistan BCG coverage, 2000–2015 as an example.

It is timely to assess vaccine target populations, using indicators to flag potential inconsistencies and inaccuracies, as the deadline for reaching the coverage targets of the Decade of Vaccines approaches. Also, in late 2015 WHO released a working draft of the Manual “Assessing and Improving the Accuracy of Target Population Estimates for Immunization Coverage”, referred to here as the *WHO Denominator Guide*, to guide program managers to assess target population estimates [3]. The *WHO Denominator Guide* recommends methods to assess accuracy such as comparing estimates with alternative sources, plotting and analyzing target populations over time, and monitoring target population growth rates, implied infant mortality rates, missing values, and consistency [3]. The *WHO Denominator Guide* also highlights how as coverage increases, the effect of a percent error in target population estimates on the error in immunization coverage estimates increases [3]. When the target population used as the denominator is underestimated, the calculated coverage is overestimated and vice versa [3]. Fig 1, which is adapted from a theoretical example in the *WHO Denominator Guide*, illustrates the effect of a 10% percent error in target population estimates on the error in coverage using Afghanistan’s rising reported administrative BCG coverage from 2000 to 2016 as an example.

This paper aims to use accuracy and consistency flags to assess the quality of target populations for Bacille Calmette-Guérin (BCG) (as pertinent based on country recommendations) and DTP3 vaccines reported by WHO Member States through the JRF between 2000 and 2016. This paper compares JRF reported target populations with UNPD LB and SI population estimates from 2000 to 2016. Applying the steps recommended in the *WHO Denominator Guide*, accuracy and consistency indicators are flagged to better understand the quality of the reported data and identify potential countries and indicators to further investigate.

## Methods

WHO Member States that use the administrative method to estimate vaccination coverage have been reporting national-level immunization coverage data for recommended vaccines to the WHO/UNICEF JRF since 1998 [5]. The JRF currently collects data on administered doses (the numerator in coverage estimates) and national target populations for each scheduled antigen (the denominator in coverage estimates) [5]. Countries that recommend the BCG vaccine typically do so at birth, so the target population is LB [3]. To account for infant mortality, the target population to administer other infant vaccines, notably DTP3, is SI [3]. Using the WHO/UNICEF JRF database as of August 2017, the target populations that countries reported for BCG and DTP3 were used as proxies for the reported estimates of LB and SI from 2000 to 2016 for 194 WHO Member States [6]. During a seventeen-year period, each annual opportunity for a country to report LB and SI through the JRF is referred to here as a reporting event. The number of doses administered for DTP3 and BCG reported through the JRF was used to calculate coverage for each year (doses administered divided by target population) [3].

The target populations reported through the JRF were compared with projected estimates of LB and SI from the UNPD 2017 revision [7]. UNPD projections were used because they are widely regarded as a “gold standard” due to the standardized, well-defined methods to develop population estimates [4]. Estimates from the UNPD 2017 revision were chosen for this analysis because they include complete population projections for all years and almost all of the countries included in this analysis, compared to previous population projections from 2002, 2004, 2012, and 2015. Additionally, the UNPD retrospectively adjusts population estimates of LB and SI with each edition, therefore the most recent UNPD edition can be assumed to be the most accurate estimate of LB and SI [7].

**Fig 2:**
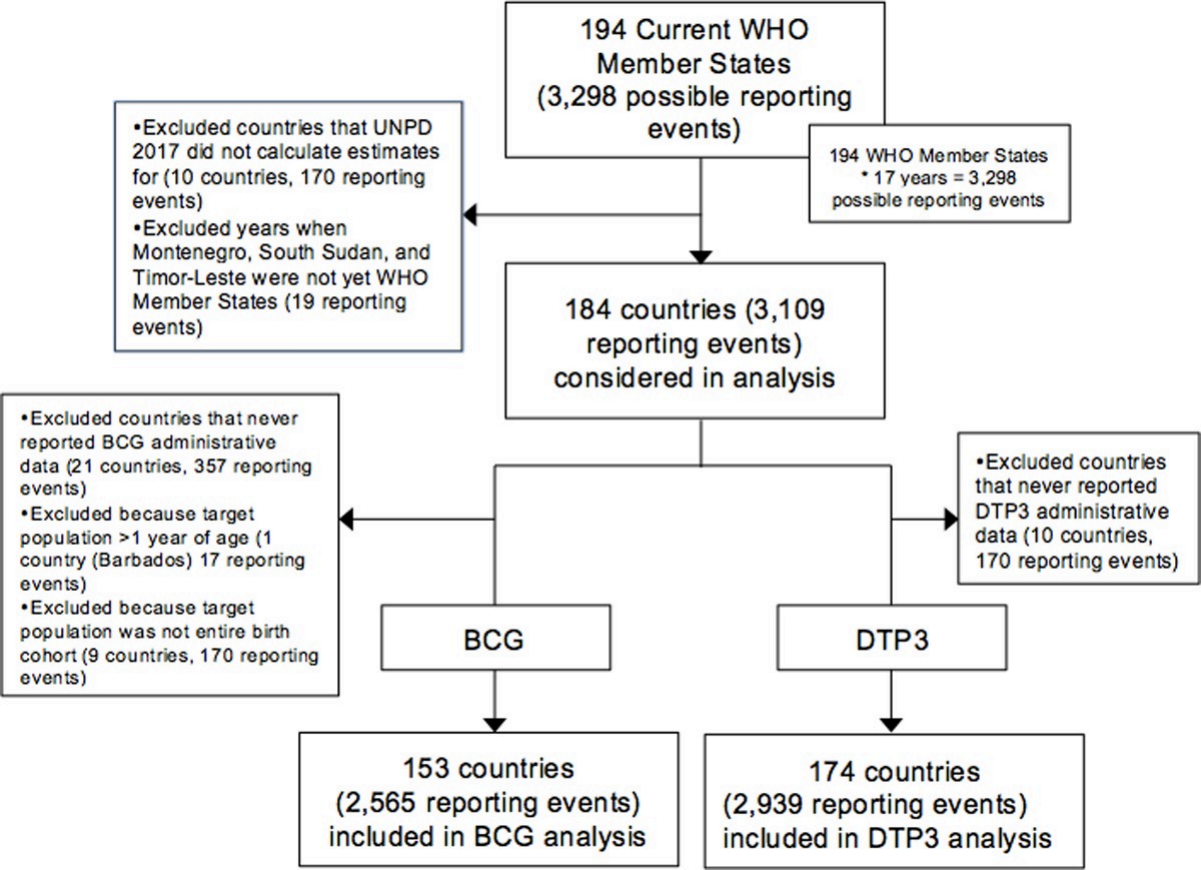
Inclusion and exclusion criteria for assessing reported BCG and DTP3 target population data from 2000 to 2016.

As of 2016, considering the 194 WHO Member States, 3,298 possible reporting events each for BCG and DTP3 were considered as the maximum possible number of reporting events for this analysis (194 countries multiplied by 17 years) (Fig 2). Montenegro, South Sudan, and Timor-Leste became WHO Members after 2000. These countries began reporting JRF data in 2006, 2011, and 2002, respectively, leading to 19 reporting events less for the analysis. Ten countries (170 reporting events) were excluded from the analysis because the UNPD 2017 did not include estimates for those countries due to their small population size. Country reporting events were excluded from the analysis of BCG when BCG was not part of the immunization schedule, the target population was not the entire LB cohort, the target population was over one year of age (only Barbados, that recommends BCG at age 5 years), [8] or data on the target population for BCG were never reported to the JRF during this time period (31 countries, 544 reporting events excluded). The analysis of DTP3 excluded countries that never reported target population data for DTP3 to the JRF during this time period and excluded reporting events when the target population for DTP3 was over one year of age (10 countries, 170 reporting events excluded). Ultimately, the JRF data included in this analysis were 153 countries and 2,565 reporting events for BCG and 174 countries and 2,939 reporting events for DTP3.

**Fig 3:**
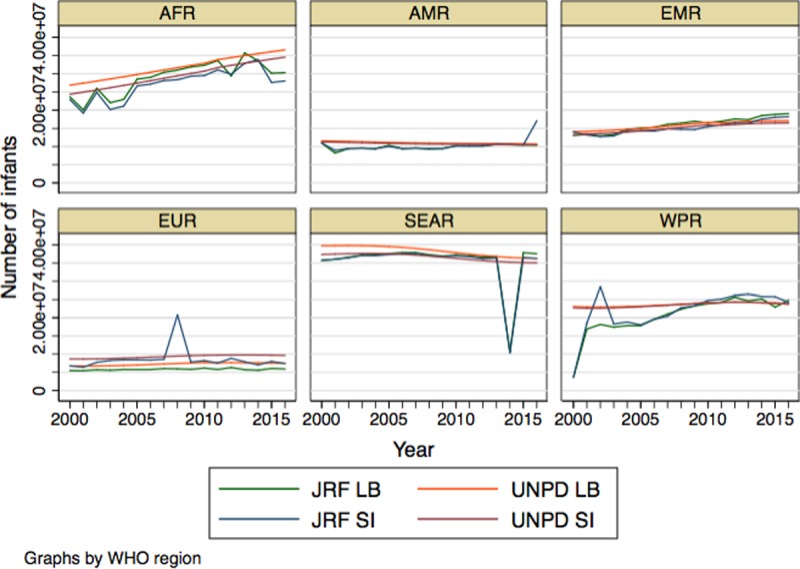
The regional number of LB and SI reported as BCG and DTP3 target populations through the JRF compared to UNPD LB and SI estimates.

Data from the 2017 UNPD were included for each country and year if the JRF data for that country and year were included in the study. There was no missing UNPD data for the countries included. The data were aggregated by sum over each of the six WHO regions (see below) to compare the total number of LB and SI reported in the JRF with the 2017 UNPD projections for each year (Fig 3).

The nine potential data inconsistencies or flags of the reported denominators included:

1. Reported LB missing;
2. Reported SI missing;
3. Year-to-year percent difference in reported LB and SI >10%;
4. Year-to-year percent difference in reported BCG and DTP3 coverage >10%;
5. Reported BCG and DTP3 coverage >100%;
6. Negative implied infant mortality rate;
7. Percent difference >10% between country BCG denominator and UNPD estimated LB;
8. Percent difference >10% between country DTP3 denominator and UNPD estimated SI;
9. Reported target population for DTP3 coverage different from that used for DTP1 coverage.

This list was selected because it includes elements related to data quality, such as completeness of reporting (1 and 2), unlikely (3 and 4) and implausible values (5 and 6), and external inconsistencies (7 and 8) [3,5]. Also, these elements have been already used to assess denominators used for immunization [3,5].

Applying calculations from the *WHO Denominator Guide*, the year-to-year percent difference in reported LB and SI for each country was calculated as: *[((Reported target population for BCG in year 2 / Reported target population for BCG in year 1)– 1) x 100]*. The same calculation was done for year-to-year percent difference in reported SI using the target population for DTP3 coverage.

Coverage of BCG and DTP3 for each country was calculated using the numerators and denominators reported in the JRF as: *[(Reported administered doses of BCG (or DTP3) in year Y / Reported target population for BCG (or DTP3) in year Y) x 100]*. Coverage greater than 100% was considered a flag of a possible underestimated target population used as the denominator.

The year-to-year percent difference in reported BCG and DTP3 coverage for each country was calculated as: *[((Coverage in year 2 / Coverage in year 1)– 1) x 100]*. Sharp year-to-year fluctuations in target populations and coverage may suggest errors in the denominators and coverage estimates [3].

The percent difference between JRF reported target populations and UNPD population estimates for each country and year were calculated as: [*(JRF reported target population for BCG in year Y–UNPD projected LB in year Y) / (UNPD projected LB in year Y) × 100]*. The same calculation was done using DTP3 and UNPD SI. An absolute percent difference greater than 10% between the target population for BCG and DTP3 from the UNPD estimates of LB and SI was another indicator of possible inaccuracy in reported target populations. The direction of the percent difference was analyzed to assess the distribution of reported target populations that were the same, less than, or more than the UNPD estimates. A negative percent difference meant that the reported target population was less than the UNPD estimate and a positive percent difference meant that the reported target population was more than the UNPD estimate.

Infant mortality rate (IMR) was calculated as: [*((Reported LB in year Y)-(Reported SI in year Y)) / (Reported LB in year Y) x 1000 children]*. Negative IMR was used as an indicator of inaccurate reported target populations because it results from having more SI in a given year than LB, which is unfeasible.

Another indicator of inaccuracy of reported DTP3 target population was whether the target population for the third dose of DTP was different from the reported target population for the first dose of DTP (DTP1) in the same year. The target population for DTP3 and DTP1 should be the same, however a different target population would require further exploration of the source of the target populations used.

For each year, the number of potential accuracy and consistency flags in a country were summed [9]. A higher number of accuracy and consistency flags in a country suggested worse quality compared to a lower number of flags.

## Results

The regional sum of LB and SI reported through the JRF compared to UNPD 2017 estimates reveal an overall trend of the reported LB and SI to be lower than the UNPD estimated LB and SI (Fig 3). The reported LB and SI in the Africa Region (AFR) show year-to-year fluctuations in reported vaccination coverage denominators. The Americas Region (AMR) displays a trend of reported LB and SI lower than the UNPD estimates in the early 2000s that gradually increases closer to the UNPD estimates over the years. The exception is in 2016, when a Latin American country reported a number of SI 916% greater than the UNPD SI estimate. The Eastern Mediterranean Region (EMR) had reported LB and SI that were generally very similar to the UNPD estimates. The European Region (EUR) overall had a trend of reporting fewer LB and SI than the UNPD estimates. The large increase in reported SI in 2008 is attributable to Russian Federation (the) reported LB 815% greater than the UNPD LB estimate. In the Southeast Asia Region (SEAR), there was a trend of reporting fewer LB and SI compared to the UNPD estimates, however this trend changed to slightly more reported LB and SI by 2015. The dramatic decrease in reported LB and SI data in 2013 is attributable to India not reporting JRF data that year. In the early 2000s, the reported LB and SI in the Western Pacific Region (WPR) had large year-to-year fluctuations and were overall lower than the UNPD estimates. The low reported LB and SI in 2000 is mostly attributable to eight out of twenty countries not reporting target population data for BCG and nine out of twenty-two countries not reporting target population data for DTP3 that year. The large increase in reported SI in 2002 is a result of Japan reporting SI 879% greater than the UNPD SI estimate. The reported WPR LB and SI gradually became more similar to the UNPD estimates, with a tendency for reported target populations to be lower than UNPD estimates. From 2011 to 2016, the WPR reported LB and SI were more similar to the UNPD estimates and slightly higher.

Out of 2,565 possible reporting events for BCG and 2,939 possible reporting events for DTP3, 194 and 274 reporting events were missing, respectively. These data were missing for reasons not attributable to the vaccine not being in the immunization schedule or the target population not meeting the inclusion criteria (Table 1). Ultimately, there were 2,371 JRF reporting events for BCG (N missing = 194 (8%)) and 2,565 UNPD reporting events for LB (N missing = 0). There were 2,665 JRF reporting events for DTP3 (N missing = 274 (9%)) and 2,939 UNPD reporting events for SI (N missing = 0). Out of the 153 countries included in the BCG analysis, 41% of countries were missing BCG data at least one year. Five countries (3%) were missing BCG target population data for 10 or more years, including: Bahamas (the), Saudi Arabia, Thailand, Trinidad and Tobago, and Turkmenistan. Of the 174 countries included in the DTP3 analysis, 47% of countries were missing target population data for at least 1 year, and seven countries (4%) were missing DPT3 data for 10 or more years, including: Czechia, Israel, Kuwait, Saudi Arabia, Slovenia, Thailand, and Turkmenistan.

**Table 1:** Summary of denominator consistency and accuracy indicators flagged, WHO Member States, 2000–2016.

Indicator		Reporting events		Countries	
		N reported data points	N observations flagged	N reported countries	N observations flagged
Reported LB missing	Any year	2565	194 (8%)	153	62 (41%)
	For 5 or more years	-	-	153	12 (8%)
	For 10 or more years	-	-	153	5 (3%)
Reported SI missing	Any year	2939	274 (9%)	174	81 (47%)
	For 5 or more years	-	-	174	20 (11%)
	For 10 or more years	-	-	174	7 (4%)
Year-to-year difference in reported LB	>5%	2158	550 (25%)	151	141 (93%)
	>10%	2158	253 (12%)	151	100 (66%)
Year-to-year difference in reported SI	>5%	2407	621 (26%)	173	162 (94%)
	>10%	2407	274 (11%)	173	120 (69%)
Year-to-year difference in reported BCG coverage	>5%	2137	788 (37%)	151	120 (79%)
	>10%	2137	423 (20%)	151	104 (69%)
Year-to-year difference in reported DTP3 coverage	>5%	2366	815 (34%)	170	144 (85%)
	>10%	2366	460 (19%)	170	116 (68%)
Reported BCG coverage >100%	Any year	2358	271 (11%)	151	66 (44%)
	For 5 or more years	-	-	151	23 (15%)
	For 10 or more years	-	-	151	8 (5%)
Reported DTP3 coverage >100%	Any year	2625	166 (6%)	172	59 (34%)
	For 5 or more years	-	-	172	11 (6%)
	For 10 or more years	-	-	172	2 (1%)
Negative implied IMR	Any year	2345	204 (9%)	153	49 (32%)
	For 5 or more years	-	-	153	17 (11%)
	For 10 or more years	-	-	153	4 (3%)
Absolute percent difference >10% between reported BCG denominator and UN LB estimate	Any year	2371	880 (37%)	153	126 (82%)
	For 5 or more years	-	-	153	81 (53%)
	For 10 or more years	-	-	153	37 (24%)
Absolute percent difference >10% between reported DTP3 denominator and UN surviving infant estimate	Any year	2665	899 (34%)	174	135 (78%)
	For 5 or more years	-	-	174	80 (46%)
	For 10 or more years	-	-	174	35 (20%)
Direction of percent different between BCG denominator and UN LB estimate	>10% Higher than UNPD Estimate	2371	372 (16%)	-	-
	>10% Lower than UNPD Estimate	2371	508 (21%)	-	-
Direction of percent different between DTP3 denominator and UN surviving infant estimate	>10% Higher than UNPD Estimate	2665	390 (15%)	-	-
	>10% Lower than UNPD Estimate	2665	509 (19%)	-	-
Reported DTP3 target population different from DTP1 target population		2416	245 (10%)	168	69 (41%)

Twelve percent of all year-to-year percent differences in reported LB exceeded 10%, with 66% of countries flagging this indicator at least once. Similarly, 11% of all year-to-year percent differences in reported SI exceeded 10%, with 69% of countries meeting this indicator at least once.

The year-to-year percent differences in reported BCG and DTP3 coverage revealed 20% and 19% of all year-to-year fluctuations exceeded 10%, respectively. Of the countries included in the analyses, 69% and 68% of countries flagged this accuracy indicator for BCG and DTP3 at least once, respectively.

Reported BCG coverage exceeded 100% in 11% of all reporting events, with 44% of countries flagging this accuracy indicator at least once. Reported DTP3 coverage exceeded 100% in 6% of all reporting events, with 34% of countries meeting this indicator at least once. Eight countries (5% of all countries) reported BCG coverage over 100% for 10 or more years, including: Argentina, Benin, Brazil, Burkina Faso, Ecuador, Ghana, Mozambique, and Nicaragua. Two countries (1% of all countries) reported DTP3 coverage over 100% for 10 or more years, including Burkina Faso and Saint Vincent and the Grenadines.

There was a negative implied infant mortality rate in 9% of all reporting events. Forty-nine countries (32% of countries) had an implied negative IMR rate at least one year. There were four countries (3% of countries) that reported a negative implied IMR for 10 or more years, including: China, Estonia, Lithuania, and the Russian Federation.

In 10% of all reporting events for DTP3, the reported DTP3 target population was different from the DTP1 target population, with 41% of countries meeting this indicator one or more years.

An absolute percent difference greater than 10% between the reported BCG target population and UNPD LB estimate was observed in 880 (37%) of all reporting events. Of the 880 observations where the LB percent difference was greater than 10%, 372 of the reported target populations were higher than the UNPD estimates and 508 reported target populations were lower than the UNPD estimates. Of the 153 countries included in the BCG analysis, 126 countries (82%) met this indicator at least one year. There were 37 countries (24%) that met this indicator for 10 or more years. Similar trends were observed in the absolute percent difference between the reported DTP3 target population and UNPD LB estimates. Overall, 899 (34%) of all reporting events had an absolute difference in SI target populations greater than 10%, of which 390 reported SI which were higher than the UNPD estimates and 509 were lower than the UNPD estimates. Of 174 countries included in the DTP3 analysis, 135 countries (78%) met this indicator at least one year and 35 countries (20%) met this indicator 10 or more years.

**Fig 4:**
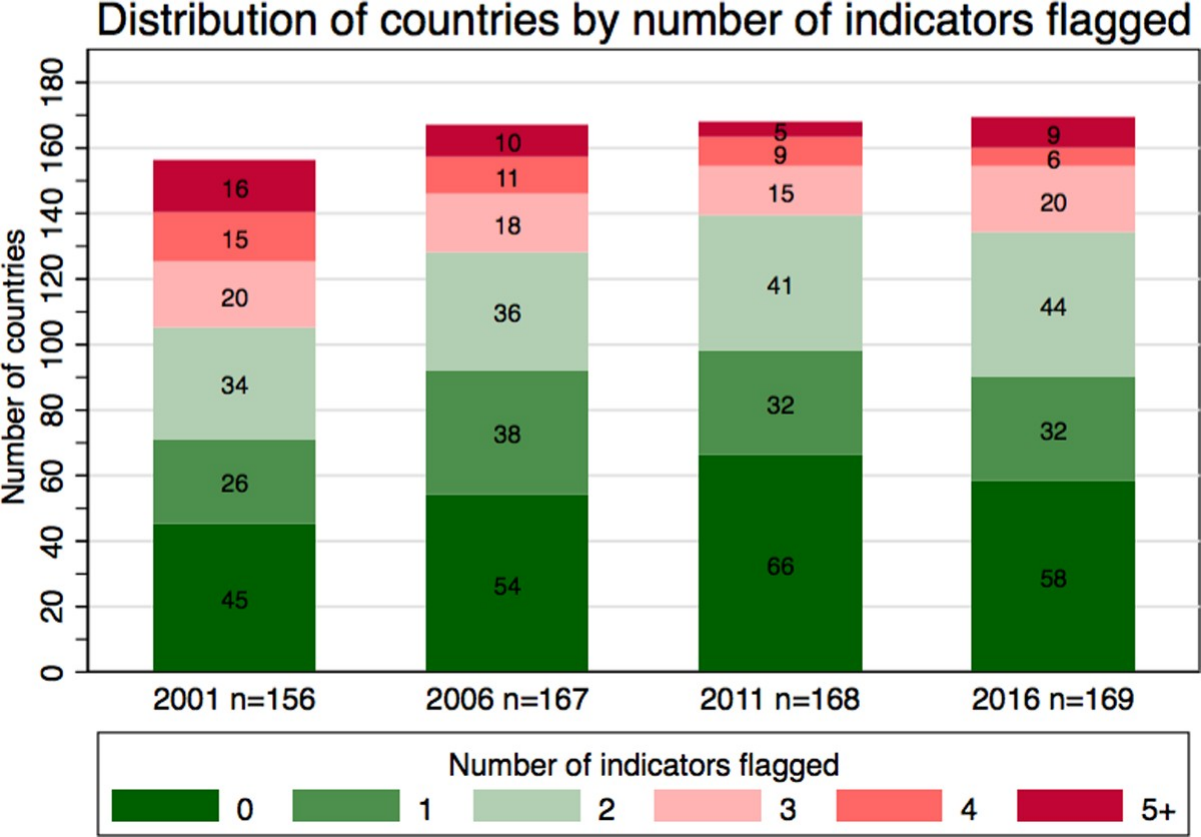
Distribution of countries by number of denominator consistency and accuracy indicators flagged over time.

When counting the number of countries that had zero through five or more indicators flagged in 2001, 2006, 2011, and 2016, there seemed to be a decrease in the number of countries that had 5 or more indicators of inconsistency or inaccuracy flagged per year (Fig 4). In 2001, there were 16 countries that flagged 5 or more of the indicators, which improved to only 9 countries in 2016. The number of countries that had zero indicators flagged increased from 45 in 2001 to 58 in 2016. It should also be noted that in 2011, there were more countries that had zero flags and less countries that had five or more flags compared to 2016. Table 2 lists the countries that met four or more inaccuracy indicators over time. The countries that are bolded are countries that appear more than once throughout the selected years.

**Table 2:** List of countries that had four or more denominator accuracy and consistency indicators flagged.

	Years			
Number of indicators	2001	2006	2011	2016
**4**	**Angola**	**Botswana**	Burundi	**Eritrea**
	Burkina Faso	Central African	**Côte d’Ivoire**	Guinea
	Cabo Verde	Republic (the)	Ecuador	**Kiribati**
	**China**	Chad	Fiji	Maldives
	**Comoros (the)**	Jordan	**Gabon**	**Nicaragua**
	**Cuba**	Kazakhstan	Guinea-Bissau	**Somalia**
	Djibouti	**Liberia**	Haiti	
	**Gabon**	**Malawi**	Latvia	
	India	Mongolia	**Nicaragua**	
	**Liberia**	Myanmar		
	Mali	Seychelles		
	Serbia	Vanuatu		
	Sierra Leone			
	South Africa			
	**Zimbabwe**			
**5**	Democratic Republic of the Congo (the)	**China**	**Botswana**	**Angola**
		**Cuba**	**Zimbabwe**	**Botswana**
		**Micronesia**		Colombia
	Ethiopia	**(Federated States**		Mauritania
	Georgia	**of)**		**Micronesia**
	Madagascar			**(Federated States**
	**Malawi**			**of)**
	Republic of Korea (the)			
	**Saint Lucia**			
	**Somalia**			
**6**	**Afghanistan**	**Comoros (the)**	**Eritrea**	**Afghanistan**
	Bhutan	**Côte d’Ivoire**	**Saint Lucia**	The United Arab
	Equatorial Guinea	Iran (Islamic	Swaziland	Emirates
	Namibia	Republic of)		Ukraine
	Papua New Guinea	**Mexico**		
	Senegal	Mozambique		
		Niger (the)		
**7**	**Kiribati**	Panama		
	**Mexico**			
**8**				Timor-Leste

Bolded countries appear more than once.

## Discussion

By applying criteria outlined in the *WHO Denominator Guide* to assess quality of reported immunization target populations, trends and patterns reveal insight into the quality of the data. The most common indicators of inaccuracy that were flagged were in reported year-to-year difference in BCG and DTP3 coverage greater than 10% and absolute percent difference greater than 10% between reported target populations and UNPD estimates. When the percent difference between reported target populations and UNPD estimates were greater than 10%, there was a tendency for the reported target populations to be lower than the UNPD estimates. This tendency to underestimate the target population in the administrative data compared to the UNPD estimates may lead to an overall overestimation of calculated coverage.

While it is difficult to quantify and fully appreciate the implications of inaccurate reported target populations on policy decisions and associated costs, if coverage is wrong, it may lead to a false sense of confidence in areas overestimating coverage and, conversely, inefficient use of resources focused on areas that are well vaccinated but underestimating their coverage. To overcome these issues, in addition to conducting periodic assessments of internal data consistency, coverage data should also be triangulated with surveillance and other related data. Triangulation for immunization has been defined “as an approach for critical synthesis of existing data from two or more independent sources to address relevant questions for program planning and decision-making. The data triangulation process identifies and aims to address limitations of any one data source and/or data collection methodology. Data triangulation also encourages deeper insight into the phenomena of interest through making sense of complementary information and integrating knowledge of the broader context and underlying process(es)” [10,11]. Stakeholders should monitor trends in reported target populations and compare reported target populations with other data sources, such as the UNPD [10,11].

Overall, there seems to be a trend of fewer countries having four or more indicators flagged, suggesting an improvement in the quality of the data. Countries in Table 2 that flagged four or more criteria in 2016 or that appear more than once (bolded countries) should assess possible reasons for this and implement strategies to improve the quality of the data if necessary. Furthermore, stakeholders could apply the methods outlined in the *WHO Denominator Guide* that were used in this analysis to gain a more country-specific understanding of trends in the quality of reported target populations. Data should be periodically assessed for outliers. For example, it is possible that an uncorrected typo was the cause of the country SI report that was 916% greater than the UNPD estimates in 2016. Stakeholders could also explore reasons for inaccuracies and inconsistencies in reported target populations to address factors that are contributing to the patterns observed and, in some cases, poor data quality. To avoid the sudden “jumps” in denominators observed here, as new data becomes available, for example new census projections, countries can consider retrospectively recalculating coverage over time using the revised target population estimates to have more meaningful coverage trends. This would ultimately guide improvements in vaccination policies, delivery, and financing.

As immunization coverage rises globally, coverage estimates become more sensitive to errors in target populations [12]. While it is not always feasible for countries to estimate target populations with high precision, analyzing the consistency and feasibility of reported target populations over time can be a cost effective way to see if immunization data has gotten “better” over the years [10]. This will be important as vaccination coverage is a key immunization indicator and is one of the Sustainable Development Goals (SDG) indicators related to healthcare [13].

It should be noted that country-specific factors affect the quality of the data. Countries may have different reporting guidelines that require coverage estimates to be based on censuses, surveys, projections, etc. Countries that use projections based on the UNPD estimates will likely have a smaller percent difference from UNPD estimates than countries that use alternative methods. For countries that estimate target populations based on projections from the most recent census, further analysis could be done correlating the time from most recent census and the quality of reported data. Year-to-year percent changes in reported LB, SI, and coverage estimates may also be explained by contextual factors, such as civil conflict. These factors may result in changes in population size and administrative capacity to report accurate data. There may also be external pressures on countries to report higher immunization coverage rates, resulting in an underreporting of target populations. There are important limitations to consider in this analysis. Countries with smaller LB and SI populations are more prone to large percent differences than countries with larger populations. Countries with larger populations also have a disproportionate impact on the sum of reported LB and SI shown in Fig 3. According to the quartiles of SI reported through the JRF, there are 31 countries that fall in the first quartile of population size (smallest), 38 in the second quartile, 36 in the third quartile, and 79 countries in the fourth quartile of SI population size (largest). The majority of countries (43%) that fall into the largest quartile of the SI population size will have a large impact on the sum of populations and be more resistant to percent differences.

When calculating the percent difference between the reported target populations and the UNPD estimates, it is assuming that the UNPD estimates are the “gold standard.” In reality, the true LB and SI of a country are unknown, so it is possible that the reported LB and SI are more accurate than the UNPD. It was out of the scope of this analysis, however future studies could investigate if the differences in LB, SI, and re-calculated coverage would change if older UNDP population projections were used.

It is possible that there are reporting events when the target population for the vaccine did not meet the inclusion criteria, for example the target population for BCG or DTP3 was not the entire LB and surviving infant cohort. Therefore, it is necessary to acknowledge that any indicators that were flagged should be further explored to understand the reason as to why. The coverage calculations are also vulnerable to inaccuracies in the numerator (the reported doses administered) for a variety of reasons. The implied IMR was calculated using the LB and SI data from the same year, so it is possible that SI would be higher than LB if the statistic included some of the birth cohort from the previous year.

As countries universally strive to increase immunization coverage as part of the Global Vaccine Action Plan and Decade of Vaccines, it is becoming all the more important to understand the quality of reported target populations. The criteria outlined in the *WHO Denominator Guide* can be a cost-effective method for countries to better understand the current status of their coverage estimates to guide immunization activities moving forward.

## Supporting information

S1 File Data used for analysis. (DOCX)
