# Supplementary material for: Assessing the quality and accuracy of national immunization program reported target population estimates from 2000 to 2016
Source: PLoS One. 2019 Jul 9;14(7):e0216933. doi: 10.1371/journal.pone.0216933 (PMC6615593; doi:10.1371/journal.pone.0216933)
Supplement: S1 File — (DOCX) [file pone.0216933.s001.docx]

The Joint Reporting Form data used for this paper was obtained from <https://www.who.int/immunization/monitoring_surveillance/data/en/> under section 4.2 “Download country reported administrative data time series.” The following variables were used:

- NamePublicationEnglish
- WHOregion
- ISO3country
- VaccineCode
  - DTP1
  - DTP3
  - BCG
- Year
- TargetGroup
- DosesAdministered
- PerCentCoverage

The United Nations Population Division projections of live births and surviving infants can be found at <https://population.un.org/wpp/Download/Standard/Interpolated/> under the excel sheet “Annual Demographic Indicators.” The 2017 population projections were used for this analysis. The following variables were used:

- Births (thousands)
- Infant deaths, both sexes combined (under age 1, thousands)
